# NETosis of psoriasis: a critical step in amplifying the inflammatory response

**DOI:** 10.3389/fimmu.2024.1374934

**Published:** 2024-08-01

**Authors:** Jinke Zhang, Yahui Feng, Dongmei Shi

**Affiliations:** ^1^ Cheeloo College of Medicine, Shandong University, Jinan, China; ^2^ The Laboratory of Medical Mycology, Jining No. 1 People’s Hospital, Jining, Shandong, China; ^3^ Department of Dermatology, Jining No.1 People’s Hospital, Jining, Shandong, China

**Keywords:** neutrophils, NEtosis, psoriasis, immune, immune disease

## Abstract

NETosis, a regulated form of neutrophil death, is crucial for host defense against pathogens. However, the release of neutrophil extracellular traps (NETs) during NETosis can have detrimental effects on surrounding tissues and contribute to the pro-inflammatory response, in addition to their role in controlling microbes. Although it is well-established that the IL-23-Th17 axis plays a key role in the pathogenesis of psoriasis, emerging evidence suggests that psoriasis, as an autoinflammatory disease, is also associated with NETosis. The purpose of this review is to provide a comprehensive understanding of the mechanisms underlying NETosis in psoriasis. It will cover topics such as the formation of NETs, immune cells involved in NETosis, and potential biomarkers as prognostic/predicting factors in psoriasis. By analyzing the intricate relationship between NETosis and psoriasis, this review also aims to identify novel possibilities targeting NETosis for the treatment of psoriasis.

## 1 Introduction

Psoriasis is a chronic immune-mediated disease that not only affects skin but is also associated with conditions such as arthritis, diabetes mellitus, metabolic syndrome, vascular complications (including stroke and ischemic heart disease), and depression (1). It is widely recognized that Th1 and Th17 and their associated cytokines, including IL-17A, IL-12, and IL-23, play a key role in the pathogenesis of psoriasis. Inhibitors targeting these cytokines have proven effective in achieving clinical remission by rapidly clearing skin lesions. However, some patients do not respond to these inhibitors or experience disease recurrence during treatment, suggesting the involvement of other immune cells in the pathogenesis of psoriasis.

Dysfunctional immune system involvement has been well-documented in typical psoriatic dermatoses, making it a focal point for investigating the pathogenesis of the disease (2). Neutrophils have been observed to be highly abundant in psoriatic lesions, particularly in the epidermis, where they accumulate in Munro micro-abscesses in the stratum corneum and spongy pustules of Kogoj in the spinous layer (3). Some researchers believe that neutrophils, as the most abundant of the innate immune cells, play a crucial role in the development and progression of psoriasis (4). Although the precise role of neutrophils in the development and progression of psoriasis remains unknown, a wealth of clinical data supports their relevance (5–7). For instance, the treatment drug for psoriasis, *dimethyl fumarate* has been shown to reduce neutrophil levels, thereby mitigating the immune system’s impact on the body. Additionally, *secukizumab*, a drug that significantly reduces epidermal neutrophil levels, has demonstrated efficacy in treating moderate-to-severe psoriasis (8). These findings suggest that neutrophils are involved in the pathogenesis of psoriasis and represent a potential target for therapeutic intervention.

Activated neutrophils employ a mechanism known as NETosis to capture and eliminate pathogens by releasing neutrophil extracellular traps (NETs) into the cell. The formation of NETs is accompanied by a unique form of neutrophil death, distinct from apoptosis and necrosis, known as NETosis (9). These NETs have been identified in peripheral tissues, such as the skin and kidneys, of individuals with autoimmune small vessel vasculitis, SLE, and rheumatoid arthritis (10–12).

In this narrative review, we extensively examine published articles focusing on the formation of NETs and their key components, as well as the potential role of NETosis in psoriasis. We also explore new treatments for psoriasis.

## 2 Overview of NETosis

### 2.1 NET formation and NET components in psoriasis

NETs, which are composed of decondensed chromatin forming a reticulated DNA structure with pores of approximately 200 nm, are surrounded by nuclear proteins (13). These proteins can be classified into three categories: histones, granule proteins, and cytoplasmic proteins. Granule proteins mainly include neutrophil elastase and myeloperoxidase. Cytoplasmic proteins include representatives such as S100 calcium-binding proteins A8, A9, and A12, as well as actin and α-actin (14–16).

The formation of NETs can involve two main mechanisms, namely vital NETs and suicidal NETs (17). Suicidal NETosis is the release of DNA networks by neutrophils through apoptosis, in which the nucleus is discharged into the surrounding environment (18). When neutrophils perceive a stimulus, the stimulus directly activates the protein kinase C (PKC) and Raf-MEK-ERK-MAP kinase pathways. Next, the activation of MAP kinase will initiate the formation of the NADPH oxidase complex (19, 20), leading to the rapid generation of reactive oxygen species (ROS) (21). Neutrophil elastase (NE) and myeloperoxidase (MPO) contribute to the enhancement of nuclear membrane permeability and the promotion of chromatin formation (22), as well as in the nucleus, where NE and MPO can facilitate the digestion of histones H2b and H4 through synergistic effects (23, 24). At the same time, ROS may increase Ca^2+^ in the cytoplasm by disrupting the endoplasmic reticulum or mitochondrial membrane, thereby activating peptide-based arginine deaminase 4 (PAD4) (25). Then, PAD4 modifies histone H3 by converting arginine to citrulline, leading to chromatin depolymerization (26). During nuclear rupture, citrulline histones (26, 27) and nuclear DNA (28)are released together. The released DNA is further decorated by granular (NE and MPO) (29) and cytosolic proteins (Calpain) (30). The increase of intracellular ROS can also activate receptor interacting protein kinase 3 (RIPK3) and mixed lineage kinase domain like protein (MLKL), promoting membrane rupture (31, 32).

Vital NETosis is formed by neutrophils passing through the cell membrane and releasing DNA and proteins into the surrounding environment to form NETs (33). At first, PMA stimulates neutrophils, leading to rapid activation of intracellular NADPH oxidase and ultimately increasing ROS (19, 34). When DNA dissociated from chromatin leaves the cytoplasm through vesicles, it can be modified by granular proteins (NE, MPO, and PR3) (29, 34). Subsequently, neutrophils maintain activity and exert further functions (35), using the increased intracellular ROS to mobilize the cytoskeleton to transport particles and mitochondria (36), and using ATP to transport particles to the outside of neutrophils through actin (37).

### 2.2 NET components in psoriasis

There is a higher likelihood of NET formation in neutrophils in individuals with psoriasis than in healthy individuals (38). Furthermore, it has been observed that there may be alternations in NETs during the onset of the disease. A recent study suggests that NETs in individuals with psoriasis exhibit an increased presence of proteins, including inflammatory mediators and antimicrobial proteins such as histone, myeloperoxidase, neutrophil elastase LL37, and RNA-LL37 (39). These proteins are believed to play a role in the inflammatory process that contributes to the development of skin lesions.

## 3 NETosis is associated with the amplification of psoriasis inflammation

NETosis, a process in which neutrophils release NETs, plays a significant role in amplifying inflammation in psoriasis. During NETosis, neutrophils produce LL37, which can bind to the P2X7 receptor on monocytes and promote RNA uptake. This RNA is then directed to intracellular compartments, triggering the activation of endosomal toll-like receptors and subsequent secretion of IL-1β, leading to inflammatory vesicle activation (40).. Additionally, LL37 promotes RNA uptake by neutrophils and facilitates its transportation to intracellular compartments, resulting in TLR induction, cytokine release, and IL-8 production and CD62L shedding upon stimulation of the neutrophils (41, 42). The released IL-8 can restimulate neutrophils (43), recruiting more neutrophils to the lesion site (2). In a study conducted by Franziska et al., it was demonstrated that NETs contain RNA, and the RNA-LL37 complex has the ability to induce the release of new NETs by neutrophils, creating a repetitive cycle of immune activation that further amplifies psoriatic inflammation (42).

Furthermore, the tyrosine phosphatase SHP2 has been found to be highly correlated with neutrophils and the development of psoriasis. Experimental results from Ding Y et al. suggested that SHP2 promotes the production of NETs and increases the expression of inflammatory cytokines associated with psoriasis through the ERK5 pathway. SHP2 predominantly increased in macrophages and acts as an IL-10 inhibitor to exacerbate psoriasis progression. It is worth noting that the inhibition of SHP2 significantly improves psoriasis-like skin inflammation in mice (44–48). In addition, molecular complexes containing the adapter molecule Act1 and SHP2 mediate autonomous IL-17R signaling, thereby accelerating and maintaining inflammation (49). Consequently, SHP2 exacerbates the progression of psoriasis, making it a potential therapeutic target for the treatment of psoriasis (50).

## 4 Immune cells and inflammatory factors associated with NETosis in psoriasis

### 4.1 Neutrophils

Psoriasis, as an inflammatory skin disease, is characterized by the infiltration of neutrophils. In response to inflammatory signals, circulating neutrophils are recruited to an inflammatory site and become activated. These activated neutrophils produce and release large amounts of ROS as part of their antimicrobial activity. Two key enzymes involved in the respiratory burst and subsequent ROS production are NADPH oxidase (NOX2) (51) and MPO (52). Research has shown that neutrophils from psoriasis patients have higher MPO and NOX2 activity, leading to increased ROS release compared with neutrophils from healthy individuals (53, 54). An imbalance in ROS production, either through overproduction or insufficient clearance of ROS, can result in oxidative-stress-related dysfunctions.

In patients with psoriasis, neutrophils are pre-activated and form NETs within psoriatic lesions. These NETs are increased in blood samples and correlate with the severity of psoriasis. NETs create a highly immunogenic environment and are involved in the initial and maintenance phases of psoriasis. They are enriched in RNA, particularly LL37. When RNA binds to LL37 and subsequently stimulates neutrophils, this can lead to the release of IL-8 and a moderate shedding of CD62L (41, 42). The data suggest that the involvement of neutrophils and their activation in psoriasis highlight their significant role in the pathogenesis of the disease.

### 4.2 Keratinocytes

One of the central features of psoriasis is the dysregulated crosstalk between keratinocytes and immune cells. Activated keratinocytes in psoriatic skin release pro-inflammation, IL-1, TNF, and IL-6. These cytokines not only amplify the inflammatory response but also induce the production of chemokines, which attract immune cells to the sites of inflammation. These cytokines activate dendritic cells, which in turn produce the cytokines IL-12 and IL-23, leading to the differentiation of TH1 and TH17 cells.

**Figure 1:**
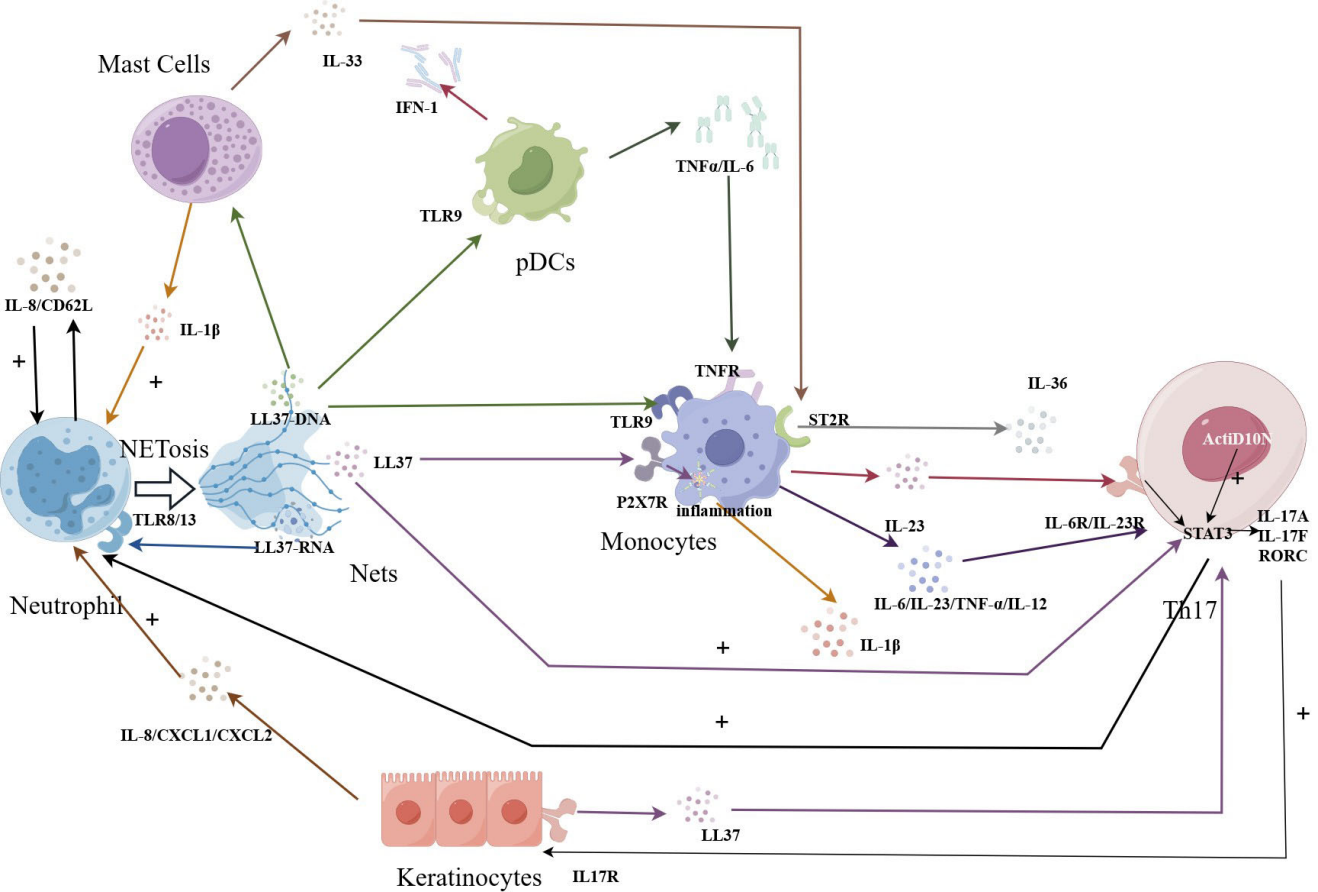
NETosis amplifies immune effects in psoriasis. The LL37-DNA causes Th17 cells to secrete cytokines, which promote keratin-forming cells to secrete LL37, which can in turn act on Th17 cells to amplify the immune effect. Beyond that, the LL37-DNA complex produced by NETosis cell can also stimulate the TLR9 receptor of PCDs, and the released TNFα binds to the receptor on monocytes. The further released IL23 can be recognized by Th17 cells. IL-17α produced by Th17 cells reactivates neutrophils, amplifying the immune and inflammatory effects.

As shown in **Figure 1**, in the context of NETosis, keratinocytes have been shown to be with NETs and their components. Studies have demonstrated that keratinocytes can internalize NETs and take up antimicrobial peptides, such as LL37 and human beta-defensin 2 (HBD2), present in these structures (55).

NETs, including psoriatic NETs, have been shown to induce HBD-2 mRNA production in epidermal keratin-forming cells, thereby promoting the expression of HBD-2 (56). Moreover, it is well-established that HBD-2 is primarily expressed in keratinocytes (57–59). In line with previous studies, Gambichler et al. reported elevated levels of HBD-2 in psoriatic skin compared with healthy controls (60). The induction of HBD-2 expression in keratinocytes by NETs suggests a potential mechanism through which neutrophils and keratinocytes contribute to the inflammatory cascade observed in psoriatic skin.

Similarly, Kanda et al. shed light on the association between LL37 levels and psoriasis. Their findings revealed significantly higher levels of LL-37 in the sera of patients with psoriasis than in normal subjects. Interestingly, the researchers also observed a correlation between serum LL-37 levels and HBD-2 levels in patients (61). HBD-2 is believed to contribute to psoriasis development by acting on neutrophils.

Taken together, the interaction between keratinocytes and neutrophils, mediated by factors such as HBD-2 and LL37, plays a role in promoting inflammation and NETosis in psoriasis. Further studies of HBD-2 may provide valuable insights into the pathogenesis of psoriasis and potential therapeutic targets.

### 4.3 Mast cells

Mast cells (MCs) play a crucial role in immune modulation through the release of various pro- and anti-inflammatory mediators (62). The role of mast cells in psoriasis has been extensively studied, with investigations indicating a significant increase in the number of mast cells at psoriatic lesions compared to healthy individuals (63). In the context of psoriasis, MCs contribute to the inflammatory state by producing caspase-1 and chymotrypsin. These enzymes play a crucial role in activating immature IL-1β into mature active IL-1β (64). The production of IL-1β by MCs further amplifies inflammation and leads to an increase in the number of infiltrating neutrophils in response to protease release (65).

Neutrophils release LL37-RNA through the NETosis process. This LL37-RNA can be recognized by MCs triggering their activation and the subsequent production of IL33. IL33 then binds to ST2R on the surface of macrophages, stimulating the production of IL-36 by macrophages.

### 4.4 DCs

Dendritic cells (DCs) are specialized antigen-presenting cells that play a crucial role in the immune system. They act as a bridge between innate and adaptive immunity by capturing, processing, and presenting antigens to T cells. They are a heterogeneous population of cells comprising different subpopulations, including plasmacytoid DCs (pDCs), classical/myeloid DCs (cDCs/mDCs), and monocyte-derived dendritic cells (moDCs) (66, 67). cDCs are a major subset of DCs specialized in presenting antigens to CD4+ helper T cells. Through this antigen presentation, effector T cells including Th2 and Th17 cells are activated (68). pDCs mainly produce a large amount of IFN-α and IFN-β, which can also directly activate T cells through stimulation (69). moDC can effectively express TNF- α and inducible nitric oxide synthase (iNOS), and iNOS-mediated NO production inhibits T cell proliferation (70).

In psoriasis, studies have shown that the number and activity of pDCs are increased in areas of skin lesions. These pDCs are responsible for producing interferon in psoriatic plaques (71). Interferon production by pDCs in psoriasis may trigger an immune response that exacerbates symptoms. The overproduction of interferon can lead to the abnormal proliferation of skin cells and inflammation, ultimately resulting in the formation of typical psoriatic lesions. Recent studies have also found that the overexpressed antimicrobial peptide LL37 in the skin of patients with psoriasis can form a new complex with self -DNA. These complexes can trigger TLR9 in dendritic cells, specifically pDCs (72, 73). When neutrophils capture pathogens and release NETs, the DNA-LL37 complexes within these NETs can be captured by dendritic cells, particularly pDCs. Dendritic cells then process these antigens and present them to T cells, initiating a specific immune response. This immune response involves the production of TNF-α and IL-6 (41, 73). These data indicate that the interaction between DCs and NETs is involved in the pathogenesis and progression of psoriasis.

When grouping DCs in the blood of psoriasis patients, it can be found that not only the skin but also the mDCs in the blood have Th1 polarization and Th1/Th17 recruitment abilities (74). This discovery provides a possible blood testing target for the diagnosis of psoriasis. In addition to directly stimulating Th1 polarization, mDCs can also co-culture with inflammatory polymorphonuclear leukocytes (PMNs) to form NETs, from which they can absorb antigens. This process potentially allows for antigen processing and presentation, indirectly stimulating Th1 polarization. Reducing mDCs can block the occurrence of NETosis. In a mouse model, mDCs activated by NETs can induce antineutrophil cytoplasmic antibody (ANCA) and autoimmune responses (75). When psoriasis patients experience renal organ damage, ANCA positivity may occur. mDCs may work together with NETs in this situation, exacerbating the progression of the disease.

### 4.5 Monocytes/macrophages

Monocytes and macrophages are key components of the immune system and play key roles in immune defense, surveillance, and self-stabilization. They are capable of phagocytosing and eliminating intracellular parasites, foreign bacteria, and mutated tumor cells, as well as their own senescent and abnormal cells. Macrophages can differentiate into different cell subpopulations, including two representative subpopulations, M1 and M2, based on the stimulation of different stimuli and the production of different cytokines.

Studies on mouse models of psoriasis have demonstrated a strong correlation between macrophages and the severity of psoriasis (76, 77). Pathological sections of skin lesions from psoriasis patients have also shown the presence of aggregated macrophages (78). When staining the skin lesions of psoriasis patients, it can be observed that CD68^+^iNOS^+^M1 increase and CD68^+^CD163^+^M2 decrease (79). Increased M1 polarization in psoriasis patients is associated with increased disease severity (80). In addition, the number of CD68^+^ (81) and CD163^+^ macrophages expressing TNF-a in the dermis (82) also increased in human skin with psoriasis lesions (82). Activation of the NLRP3 inflammasome by macrophages can also be involved in psoriasis (83). Research has shown that NLRP3 may be a promising therapeutic target for the treatment of psoriasis (84). The method of inhibiting NLRP3 inflammasome activation can alleviate psoriasis inflammation (85).

In this context, when dying neutrophils release LL37 through NETosis, the P2X7 receptor on monocytes is activated. This activation triggers the release of inflammatory vesicles and the production of IL-1β (32, 33). Similarly, when LL37-DNA complexes are released, the TLR9 receptor on monocytes is activated, leading to the release of IL-6, IL-12, IL-23, and TNF-α (20). These cytokines, including those produced by pDCs, can further stimulate monocytes/macrophages to produce IL-23. The accumulation of IL-23 at the site of a skin lesion can lead to the production of additional cytokines by macrophages, including IL-17A, IL-22, and IFN-γ, in addition to TNF-α (86).

### 4.6 Th17 cells

It is widely recognized that IL-23 plays a crucial role in maintaining the activation of Th17 cells (87). IL-23 promotes the production of IL-17A by Th17 cells, which in turn leads to the recruitment and activation of neutrophils (88). This cytokine cascade contributes to the inflammatory response observed in psoriasis. In *in vitro* experiments, the percentage of CD3+CD4+IL-17+ (Th17) cells among T cells is significantly higher in the presence of NETs compared to the control group without NETs. Act1 is a key mediator for IL-17 signal transduction (89). In the presence of NETs, the downstream key factor Act1D10N of the psoriasis susceptibility gene *TRAF3IP2* mutation is enhanced, further inducing the production of Th17 cells (90). In summary, these results indicate that NETs are of great significance in the immunogenetic study of neutrophil-induced human Th17 cells and psoriasis.

Studies have indicated that NETs are abundant in environments rich in myeloid cells and memory T cells. This suggests that NETS play a role in inducing the formation of other immune cells (91, 92). Experimental findings by Evans et al. support this notion, demonstrating a link between NETs and Th17 responses in psoriasis patients. The researchers further explored this interaction, showing that NETs can induce the differentiation of memory CD4 T cells into LL37-specific Th17 cells (93). These memory T cells not only secrete IL-17A but also express IL-17F and RORC, which stimulates keratinocytes to secrete LL37 (90). Subsequently, IL-6/IL-23 secreted by monocytes induces LL37-specific Th17 cells to migrate to the epidermis, where they recognize the LL37 expressed by keratinocytes (94). This mechanism creates an immune amplification effect. Th17 cells identify the synthesis of LL37 as a T-cell antigen, and their responses are further fueled by a synergistic interaction of IL-1, IL-6, and IL-23 (34, 35).

## 5 NET markers as prognostic/predicting factors in psoriasis

MicroRNAs (miRNAs) are small non-coding RNAs with important roles in post-transcriptional gene expression. Deregulation of miRNAs and the corresponding target gene expression have been shown to be involved in psoriasis (95). Pathogenesis MiRNA-155 (96, 97), 210 (98), and 20b (99) are significantly increased in psoriasis lesions. Among them, the expression of miRNA-155 is increased in diseased psoriasis skin compared with normal skin (100). The pathological miRNA-210 is positively correlated with the Psoriasis Area and Severity Index (PASI) and body surface area (BSA) affected by psoriasis (99). MiRNA pathway enrichment and target gene network analysis were performed on the serum of psoriasis patients, and researchers found a high correlation between miR-214–3p, miR-7–5p, miR-761, miR-665, and miR-1207–5p (101). The above results indicate an important relationship between this miRNA and disease activity, and may encourage further studies to explore the possibility of using this miRNA as one of the markers of psoriasis severity.

Studies have shown that circulating MPO/DNA or NE/DNA conjugates, as well as plasma circulating citrullinated histone H3 (H3Cit) levels, have a stronger specificity for NET formation than evaluating microRNAs alone (102). A study was conducted on the sera of 50 adult patients with chronic plaque psoriasis and 25 healthy controls, and it was found that there was a significant difference in serum myeloperoxidase levels between the two groups (103). MPO-DNA complex level is also an important detection method. It has been reported that the MPO-DNA complex level in serum was significantly increased in patients with PsA/PsO compared with healthy controls. The level of MPO-DNA was also positively associated with the Disease Activity in Psoriatic Arthritis score (DAPAS) and its components (104). Vascular endothelial cells play an important role in maintaining the vascular barrier and controlling blood flow. Additionally, they can target immune cells to specific areas of vascular damage, infection, or foreign objects (105). According to one report, H3Cit can directly cause inflammatory damage by disrupting the microvascular endothelial barrier (106). However, currently, there are no clinical data to prove the association between H3Cit and psoriasis. Whether H3Cit can become a diagnostic marker in the blood of psoriasis patients deserves further research.

Some data suggest that circulating NETs may play a role in predicting the severity of psoriasis. However, owing to the lack of specific antibodies for NETs and specific and standardized testing methods for NETs at present, NET substitutes are usually used. These detectable alternatives include circulating cell-free DNA (cfDNA), or circulating NET-associated proteins such as NE or MPO, and/or levels of circulating histone H3 (H3Cit) or other NET-associated proteins (107). Therefore, further research and technological improvements are needed to better define the prognosis and/or predictive ability of NETs at different stages of psoriasis.

## 6 The potential of drugs targeting NETosis in psoriasis

Exploring potential drugs targeting NETosis holds great promise for individuals with psoriasis, considering the involvement of NETosis in the condition. NETs inhibitors can target various stages of NETosis to inhibit the generation of NETs. For example, researchers have focused on the stress response protein REDD1, which is closely related to NETosis. Key mediators, such as endothelin-1 (ET-1) and hypoxia inducible factor-1α (HIF-1α), drive the generation of NETs through REDD1. Inhibitors like *bosentan* and *L-ascorbic acid* can respectively inhibit ET-1 and HIF-1α, thereby inhibiting NETosis in neutrophils (108).

Another crucial enzyme involved in NET formation is a protein arginine deiminase, PAD4, which catalyzes the conversion of arginine to citrulline and mediates NET formation. Drugs targeting PAD4, such as *JBI-589* (109), have shown efficacy in rheumatoid arthritis mouse models. Additionally, drugs like *dipyridamole* (110) and *cannabidiol* (111) can inhibit NETosis and have potential in the treatment of psoriasis.

Neutrophil elastase inhibitor *sivelepristal sodium* (112) and myeloperoxidase inhibitor *PF-1355* (113) have demonstrated effectiveness in inhibiting NET formation, making them potential treatments for acute respiratory distress syndrome (ARDS) or systemic inflammatory response syndrome (SIRS) with acute lung injury (ALI) (114). *Metformin* commonly used as a first-line drug for the treatment of type 2 diabetes, has also shown potential in downregulating the generation of NETs and reducing the release of NET DNA in the mouse model of systemic lupus erythematosus (SLE) (115). However, there is a need for relevant clinical and *in vivo* experiments to determine their effectiveness in psoriasis.

**Figure 2:**
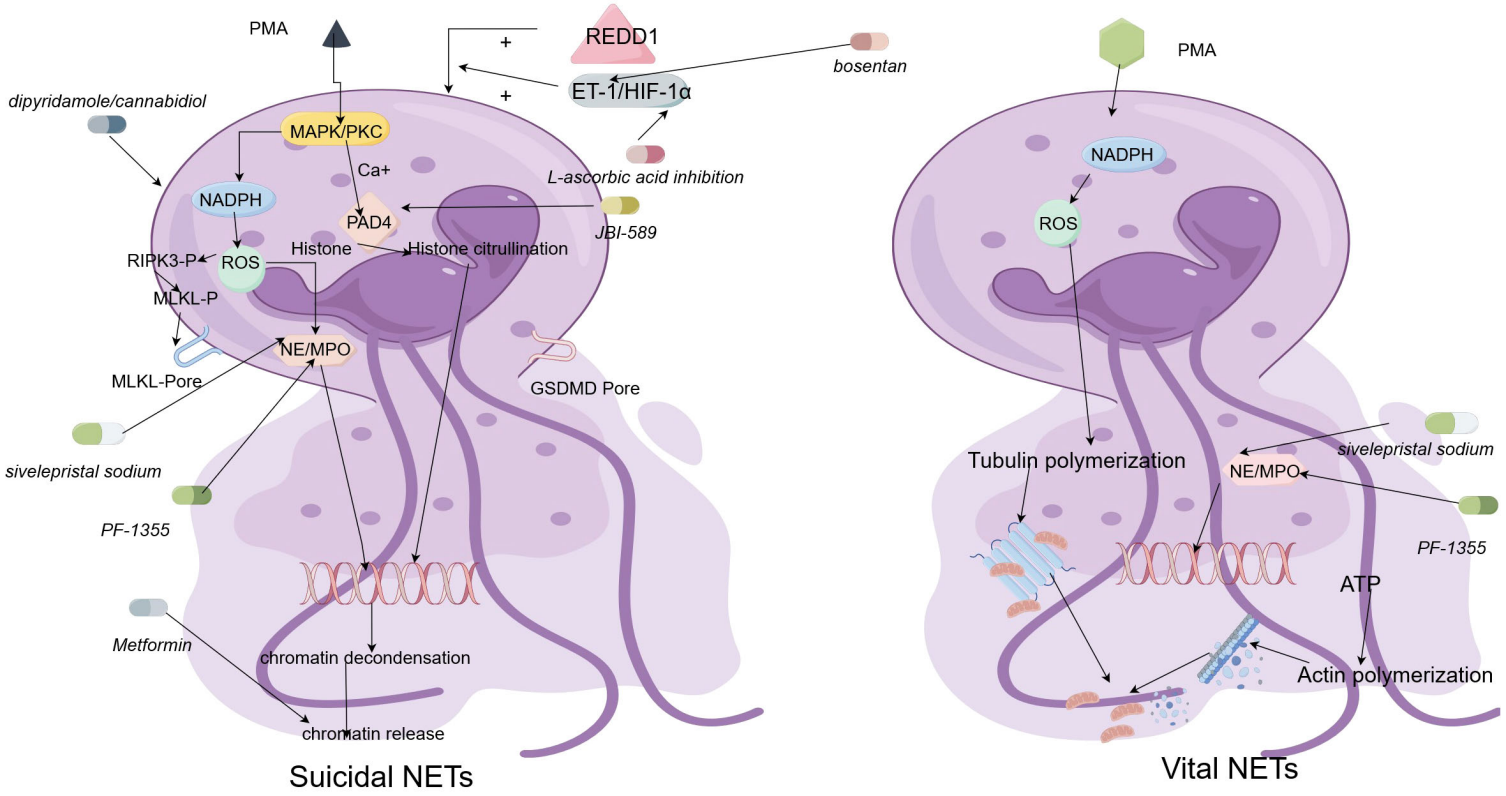
The regulatory mechanisms underlying the formation of vital neutrophil extracellular traps (Vital NETs) and suicidal neutrophil extracellular traps (Suicidal NETs), as well as a schematic diagram of the mechanism of action of NETosis drugs. The characteristics of suicidal NETosis are the production of ROS and the rupture of neutrophils. Neutrophils are stimulated and activated, inducing the phosphorylation of NOX complexes and release of ROS, a process dependent on high Ca2+concentrations. Subsequently, PAD4 is activated and causes NE and MPO to be transported from neutrophilic granules to the nucleus. NE and MPO binding to PAD4 leads to histone citrullination and chromatin deconcentration. After the nuclear membrane ruptures, the desorbed chromatin mixes with granular proteins and enters the cytoplasm. Finally, the cytoplasmic membrane leaks, and the modified chromatin is released from neutrophils, forming NETs. The formation of Vital NET can occur without NOX complexes and ROS. The formation of Vital NET is initiated by stimulation, which activates PAD4 and transports NE and MPO to the nucleus, promoting chromatin deconcentration. Decondensed chromatin decorated with granular proteins and histones is enveloped in vesicles germinating from the nucleus. Subsequently, these vesicles are expelled from intact neutrophils and form NETs near the neutrophils. Under this method, neutrophils remain intact and can further phagocytose. NETosis-related drugs can exert effects on the occurrence process of these two types of NETosis.

Clearing NETs and preventing their accumulation in the body is another approach worth considering. *Deoxyribonuclease I* (*DNase I*) (116) has been shown to effectively clear NETs in experiments. Although it can promote inflammation resolution and reduce the accumulation of ROS, it has the disadvantage of a short action time and limited range of action. In 2021, Xin’s team developed a new nanocarrier that can release DNase I in response to MMP-9, effectively degrading the structure of NETs (117). This carrier successfully addresses the drawbacks of DNase I drugs. *Tofacitinib* (118), another type of NET scavenger, can simultaneously regulate the formation and degradation of NETs. A clinical trial conducted in SLE patients showed that *Tofacitinib* can reduce low-density granulocytes and circulating NETs, indicating its potential for treating psoriasis (119). **Figure 2** provides a brief summary of these possible NETosis drugs.

In summary, drug development targeting NETosis and the interactions between NETs and various immune cells holds great promise. However, it is important to consider that NETosis also serves as a mechanism to trap and kill bacteria and other pathogens. In individuals with psoriasis, skin inflammation is associated with bacterial infection, and NETosis may be a means through which neutrophils fight infection. Furthermore, the adverse reactions and success rates of new drugs are worth further discussion.

## 7 Conclusion

Understanding the role of NETosis in the pathogenesis of psoriasis could provide insights into potential therapeutic strategies. NETosis, psoriasis, and the immune response are interconnected and closely related. Recent advancements in understanding NETosis have the potential to improve our comprehension of the complex process of psoriasis pathogenesis. This interaction provides new insights into the molecular mechanisms underlying the disease. The regulation of NETosis is rapidly emerging as a promising therapeutic target for psoriasis. Studying the exact mechanisms of NETosis in psoriasis is of significant importance for developing novel therapeutic approaches.

## Author contributions

JZ: Data curation, Writing – original draft. YF: Investigation, Writing – review & editing. DS: Writing – review & editing, Funding acquisition.
